# Na_3_Al(AsO_4_)_2_

**DOI:** 10.1107/S1600536813002213

**Published:** 2013-01-31

**Authors:** Noura Fakhar Bourguiba, Mohamed Faouzi Zid, Ahmed Driss

**Affiliations:** a Laboratoire de Matériaux et Cristallochimie, Faculté des Sciences de Tunis, Université de Tunis ElManar, 2092 ElManar II Tunis, Tunisia

## Abstract

The structure of the title compound tris­odium aluminium bis­(arsenate), Na_3_Al(AsO_4_)_2_, is built up from AlO_4_ and AsO_4_ corner-sharing tetra­hedra, forming an undulating two-dimensional framework parallel to (100). The layers are constituted of large Al_6_As_6_O_36_ rings made up from six AlO_4_ and AsO_4_ tetra­hedra in which two sodium cations are situated, the third sodium cation being located in the inter­layer space. The structural relationships between the title compound and Na_3_Fe(PO_4_)_2_, NaAlCo(PO_4_)_2_ and Al_5_Co_3_(PO_4_)_8_ are discussed.

## Related literature

For open-framework structures, see: Colomban (1986 ▶); Brunet *et al.* (2003 ▶); Alamo & Roy (1984 ▶); Delmas *et al.* (1987 ▶). For details of the preparation, see: Driss & Jouini (1994 ▶); Masquelier *et al.* (1995 ▶). For closely related structures, see: Belkhiria *et al.* (1998 ▶); Chen *et al.* (1997 ▶); Bennet & Marcus (1988 ▶). For bond-valence calculations, see: Brown & Altermatt (1985 ▶).

## Experimental

### 

#### Crystal data

Na_3_Al(AsO_4_)_2_
*M* *_r_* = 373.79
Monoclinic, 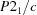

*a* = 6.5948 (7) Å
*b* = 11.8018 (9) Å
*c* = 9.4692 (8) Å
β = 96.52 (1)°
*V* = 732.23 (12) Å^3^
*Z* = 4
Mo *K*α radiation
μ = 9.44 mm^−1^
*T* = 298 K
0.28 × 0.18 × 0.12 mm

#### Data collection

Enraf–Nonius CAD-4 diffractometer
Absorption correction: ψ scan (North *et al.*, 1968 ▶) *T*_min_ = 0.15, *T*_max_ = 0.32
2484 measured reflections
1596 independent reflections
1484 reflections with *I* > 2σ(*I*)
*R*_int_ = 0.032
2 standard reflections every 120 min intensity decay: 1.2%

#### Refinement

*R*[*F* ^2^ > 2σ(*F* ^2^)] = 0.022
*wR*(*F* ^2^) = 0.058
*S* = 1.09
1596 reflections
128 parameters
Δρ_max_ = 0.81 e Å^−3^
Δρ_min_ = −1.02 e Å^−3^

### 

Data collection: *CAD-4 EXPRESS* (Duisenberg, 1992 ▶; Macíček & Yordanov, 1992 ▶); cell refinement: *CAD-4 EXPRESS*; data reduction: *XCAD4* (Harms & Wocadlo, 1995 ▶); program(s) used to solve structure: *SHELXS97* (Sheldrick, 2008 ▶); program(s) used to refine structure: *SHELXL97* (Sheldrick, 2008 ▶); molecular graphics: *DIAMOND* (Brandenburg, 1998 ▶); software used to prepare material for publication: *WinGX* publication routines (Farrugia, 2012 ▶).

## Supplementary Material

Crystal structure: contains datablock(s) I. DOI: 10.1107/S1600536813002213/vn2065sup1.cif

Structure factors: contains datablock(s) I. DOI: 10.1107/S1600536813002213/vn2065Isup2.hkl

Additional supplementary materials:  crystallographic information; 3D view; checkCIF report

## Figures and Tables

**Table 1:** Selected bond lengths (Å)

Al1—O2^i^	1.771 (2)
Al1—O4	1.755 (2)
Al1—O6^ii^	1.769 (2)
Al1—O8^iii^	1.755 (2)
As1—O1	1.654 (2)
As1—O2	1.732 (2)
As1—O5^iv^	1.649 (2)
As2—O3	1.655 (2)
As2—O4^v^	1.723 (2)
As2—O6	1.734 (2)
As2—O7	1.655 (2)

Symmetry codes: (i) 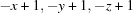; (ii) 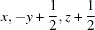; (iii) 
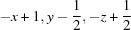
; (iv) 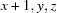; (v) 
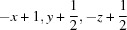
.

**Fig. 1.**
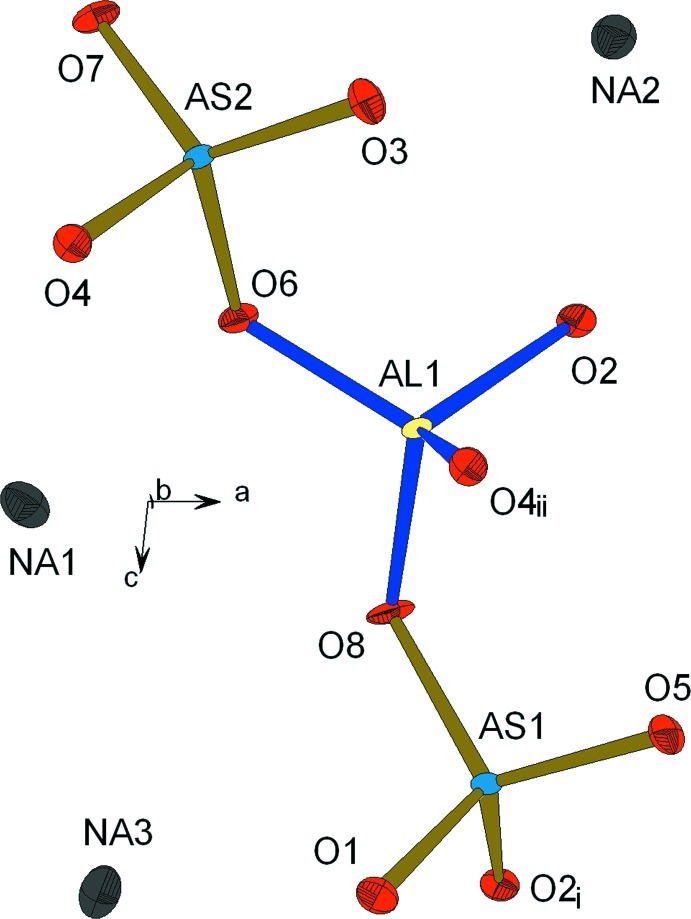
Unité asymétrique dans Na3Al(AsO4)2. Les ellipsoïdes ont été définis avec 50% de probabilités. [codes de symétrie:(i) x, 1/2 - y, 1/2 + z; (ii) 2 - x, 2 - y, 1 - z].

**Fig. 2.**
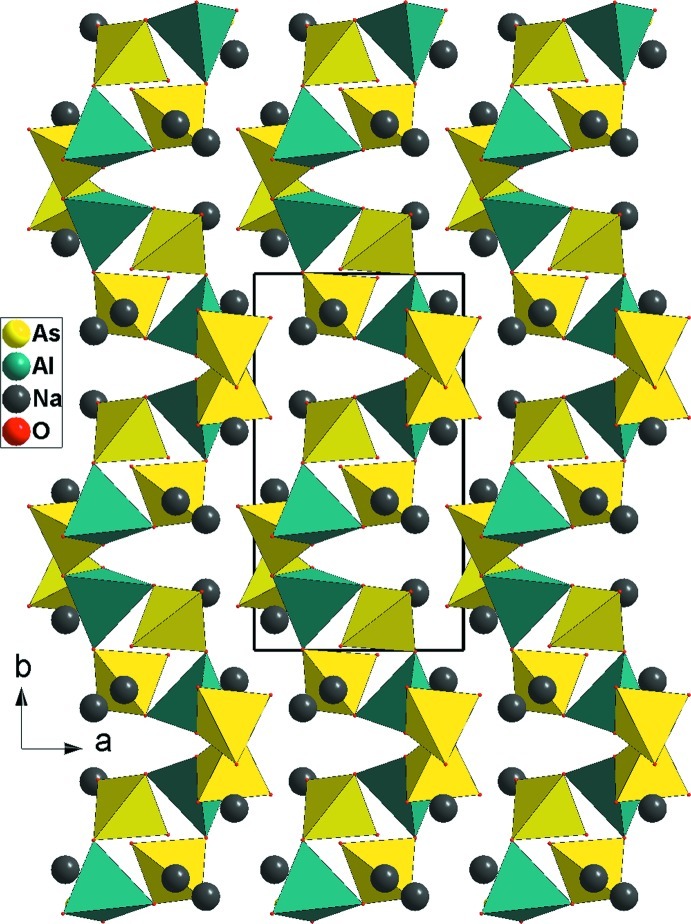
Projection de la structure selon la direction [001] montrant les couches ondulées disposées selon le plan (100).

**Fig. 3.**
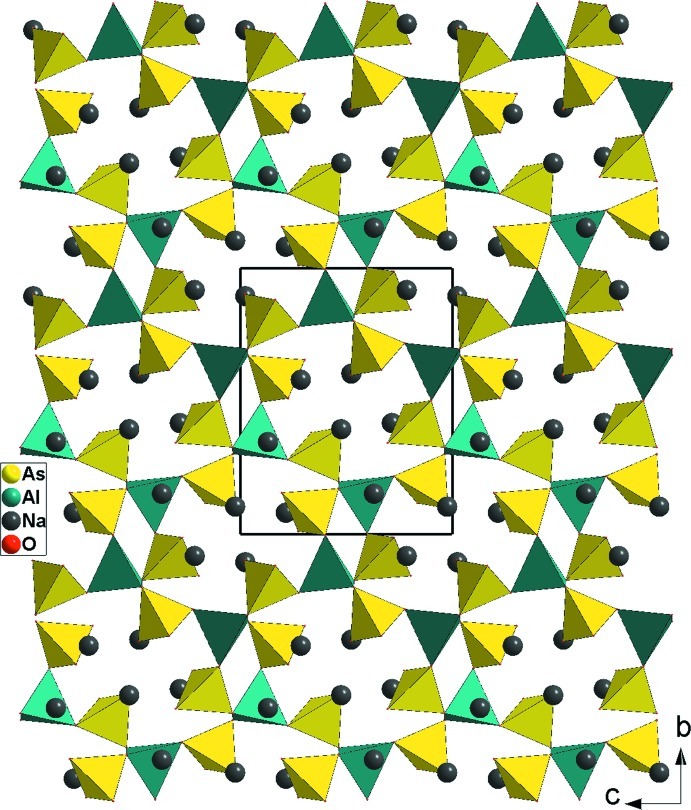
Vue de la structure selon [100] montrant les différents cycles Al6As6O36 où sont disposés les cations excentrés.

**Fig. 4.**
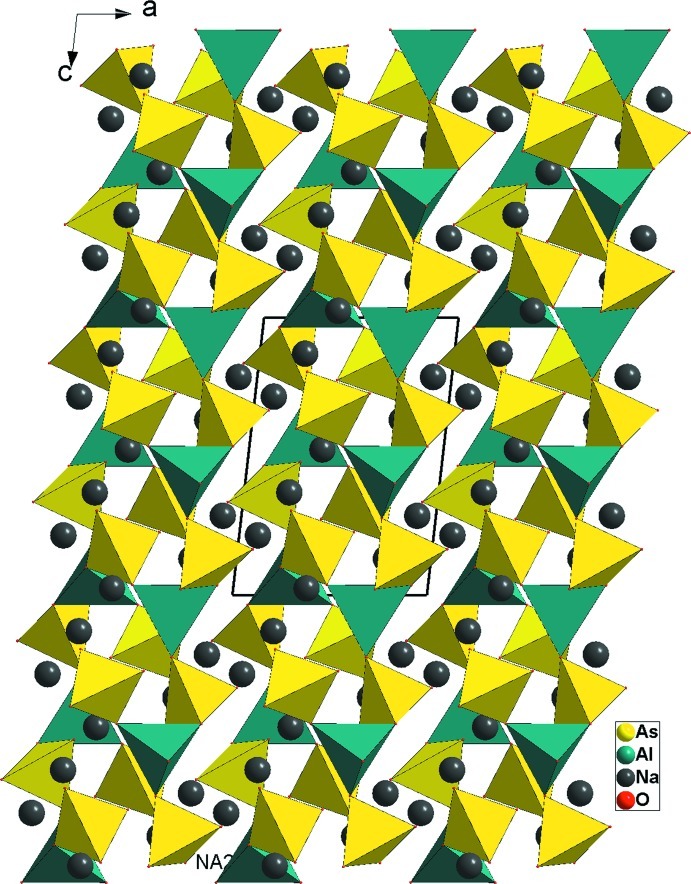
Projection de la structure de Na3Al(AsO4)2 selon [010] montrant l'espace intercouches où sont logés les cations Na2.

## supplementary crystallographic information

### Comment

Les phosphates et les arséniates mixtes de métaux trivalents et des alcalins présentent de nombreuses propriétés physico-chimiques en relation avec leurs structures. La famille des composés de type *M*'*M*(XO_4_)_3_ (*M*'=alcalin; *M*=Al, Fe, Cr, Ga, *etc*; *X*=P, As) de structure nasicon sont des matériaux attractifs dans des domaines variés de la chimie des solides: conductivité ionique, céramique à faible coefficient de dilatation thermique et intercalation (Colomban, 1986; Brunet *et al.*, 2003; Alamo *et al.*, 1984; Delmas *et al.*, 1987). Dans ce cadre et en vue de synthétiser de nouveaux matériaux à charpentes ouvertes, nous avons entrepris l'exploration du système Na–Al–As–O dans lequel différentes phases ont été isolées: NaAl_2_As_2_O_7_ (Driss *et al.*, 1994), Na_3_Al_2_(AsO_4_)_3_ (Masquelier *et al.*, 1995). Une nouvelle phase de formulation Na_3_Al(AsO_4_)_2_ a été synthétisée par réaction à l'état solide. L'unité asymétrique, dans le composé étudié, renferme un tétraèdre AlO_4_ lié par mise en commun de sommets à deux pyramides AsO_4_ (Fig. 1). Il en résulte l'unité linéaire AlAs_2_O_10_. La jonction de ces dernières, par formation de ponts mixtes Al–O–As, conduit vers des couches ondulées parallèles au plan (100) (Fig. 2). En effet, l'examen de ces feuillets révèle la présence de larges cycles de formulation Al_6_As_6_O_36_ constitués par six pyramides AsO_4_ et six AlO_4_ (Fig 3). De plus, on peut signaler qu'au sein d'une couche, chaque tétraèdre AlO_4_ partage tous ses sommets oxygène avec respectivement quatre tétraèdres AsO_4_ différents. Cependant une pyramide AsO_4_ n'engage que deux de ses sommets dans les ponts mixtes As–O–Al. Les deux autres restant libres sont orientés vers l'espace intercouches où résident les cations Na^+^ (Fig. 4). L'examen des facteurs géométriques dans la structure montre qu'ils sont conformes à ceux rencontrés dans la littérature (Driss *et al.*, 1994; Masquelier *et al.*, 1995). De plus, l'utilisation de la méthode BVS, pour le calcul des différentes valences des liaisons, en utilisant la formule empirique de Brown (Brown & Altermatt, 1985) vérifie bien les valeurs attendues de charges des ions: Al1(2,846), As1(4,971), As2(4,923), Na1(1,069), Na2(1,038) et Na3(1,027). La comparaison de la structure de Na_3_Al(AsO_4_)_2_ avec celle rencontrée dans la littérature et de formulation analogue Na_3_Fe(PO_4_)_2_ (Belkhiria *et al.*, 1998) révèle une différence nette dans la disposition des polyèdres. En effet, on note que la structure de l'arséniate est formée uniquement par un assemblage de tétraèdres AsO_4_ et AlO_4_ alors que celle du phosphate de fer est constituée par une succession de couches parallèles formées d'octaèdres FeO_6_ et de tétraèdres AsO_4_ partageant des sommets. Par contre, la comparaison de la structure étudiée avec celles des phosphates de cobalt de formulation NaAlCo(PO_4_)_2_ (Chen *et al.*, 1997) et Al_5_Co_3_(PO_4_)_8_ (Bennet *et al.*, 1988), montre des points de ressemblance. En effet, dans chacune des structures les couches sont formées uniquement par des tétraèdres (As/P)O_4_ et (Al/Co)O_4_ mettant en commun des sommets. Cependant, ces structures présentent des différences: dans notre composé, on distingue que la connexion entre couches est assurée par les polyèdres de cations alcalins conduisant à une structure bidimensionnelle. Par contre, dans les composés NaAlCo(PO_4_)_2_ et Al_5_Co_3_(PO_4_)_8_, on remarque que la connexion entre les couches est réalisée uniquement par ponts (As/P)–O–(Al/Co) permettant de décrire la structure comme une structure tridimensionnelle. Dans notre composé, seuls les tétraèdres AsO_4_ engagent tous leurs sommets dans des liaisons de type As–O–Al, alors que les tétraèdres AlO_4_ n'engagent que deux de leurs sommets dans ce type de liaisons. Cependant, dans les composés NaAlCo(PO_4_)_2_ et Al_5_Co_3_(PO_4_)_8_, tous les tétraèdres PO_4_ et (Al/Co)O_4_ engagent tous leurs sommetes dans les ponts mixtes P–O–(Al/Co).

### Experimental

Les cristaux relatifs à la phase Na_3_Al(AsO_4_)_2_ ont été obtenus à partir des réactifs: NaHCO_3_ (Prolabo, 27778), Al_2_O_3_ (Riedel-De Haen, 167305), NH_4_H_2_AsO_4_ (préparé au laboratoire, JCPDS-775), pris dans les proportions molaires: Na:Al:As=5:1:3. Le mélange, finement broyé, a été mis dans un creuset en porcelaine, placé dans un four puis préchauffé à l'air à 623 K pendant 24 heures en vue d'éliminer les composés volatils. Il est ensuite porté, par palier de 100 degrés suivi de broyage, jusqu'à une température de synthèse proche de la fusion, 973 K. Le mélange est alors abandonné à cette température pendant une semaine pour favoriser la germination et la croissance des cristaux. Le résidu final a subi en premier un refroidissement lent (5°/demi journée, à 873 K) puis un second rapide (50°/h) jusqu'à la température ambiante. Des cristaux transparents, de forme prismatique, de contour net et de taille suffisante pour les mesures des intensités, ont été séparés du flux par des lavages successifs à l'eau chaude.

### Refinement

La collecte a été effectuée dans le système monoclinique de groupe d'espace *P*2_1_/a. L'affinement a été conduit sans problème. Une transformation vers le groupe conventionnel *P*2_1_/c s'avère nécessaire avant l'affinement final. À la fin de l'affinement structural un examen de la Fourier différence finale ne révèle la présence d'aucun pic d'intensité significative. L'affinement de tous les facteurs thermiques anisotropes conduit à des ellipsoïdes bien définis. De plus, les densités d'électrons maximum et minimum restants dans la Fourier différence sont acceptables et sont situées respectivement à 0,86 Å de As1 et à 0,87 Å de As2.

### Crystal data

**Table:**

Na_3_Al(AsO_4_)_2_	*F*(000) = 704
*M**_r_* = 373.79	*D*_x_ = 3.391 Mg m^−^^3^
Monoclinic, *P*2_1_/*c*	Mo *K*α radiation, λ = 0.71073 Å
Hall symbol: -P 2ybc	Cell parameters from 25 reflections
*a* = 6.5948 (7) Å	θ = 10–15°
*b* = 11.8018 (9) Å	µ = 9.44 mm^−^^1^
*c* = 9.4692 (8) Å	*T* = 298 K
β = 96.52 (1)°	Prism, colourless
*V* = 732.23 (12) Å^3^	0.28 × 0.18 × 0.12 mm
*Z* = 4	

### Data collection

**Table:**

Enraf–Nonius CAD-4 diffractometer	1484 reflections with *I* > 2σ(*I*)
Radiation source: fine-focus sealed tube	*R*_int_ = 0.032
Graphite monochromator	θ_max_ = 27.0°, θ_min_ = 2.8°
ω/2θ scans	*h* = −8→2
Absorption correction: ψ scan (North *et al.*, 1968)	*k* = −15→1
*T*_min_ = 0.15, *T*_max_ = 0.32	*l* = −12→12
2484 measured reflections	2 standard reflections every 120 min
1596 independent reflections	intensity decay: 1.2%

### Refinement

**Table:**

Refinement on *F*^2^	Primary atom site location: structure-invariant direct methods
Least-squares matrix: full	Secondary atom site location: difference Fourier map
*R*[*F*^2^ > 2σ(*F*^2^)] = 0.022	*w* = 1/[σ^2^(*F*_o_^2^) + (0.0289*P*)^2^ + 1.1813*P*] where *P* = (*F*_o_^2^ + 2*F*_c_^2^)/3
*wR*(*F*^2^) = 0.058	(Δ/σ)_max_ = 0.001
*S* = 1.09	Δρ_max_ = 0.81 e Å^−^^3^
1596 reflections	Δρ_min_ = −1.02 e Å^−^^3^
128 parameters	Extinction correction: *SHELXL*, Fc^*^=kFc[1+0.001xFc^2^λ^3^/sin(2θ)]^-1/4^
0 restraints	Extinction coefficient: 0.0400 (12)

### Special details

**Table:**

Geometry. All e.s.d.'s (except the e.s.d. in the dihedral angle between two l.s. planes) are estimated using the full covariance matrix. The cell e.s.d.'s are taken into account individually in the estimation of e.s.d.'s in distances, angles and torsion angles; correlations between e.s.d.'s in cell parameters are only used when they are defined by crystal symmetry. An approximate (isotropic) treatment of cell e.s.d.'s is used for estimating e.s.d.'s involving l.s. planes.
Refinement. Refinement of *F*^2^ against ALL reflections. The weighted *R*-factor *wR* and goodness of fit *S* are based on *F*^2^, conventional *R*-factors *R* are based on *F*, with *F* set to zero for negative *F*^2^. The threshold expression of *F*^2^ > σ(*F*^2^) is used only for calculating *R*-factors(gt) *etc*. and is not relevant to the choice of reflections for refinement. *R*-factors based on *F*^2^ are statistically about twice as large as those based on *F*, and *R*- factors based on ALL data will be even larger.

### Fractional atomic coordinates and isotropic or equivalent isotropic displacement parameters (Å^2^)

**Table:**

	*x*	*y*	*z*	*U*_iso_*/*U*_eq_	
As1	0.86449 (4)	0.67433 (2)	0.37764 (3)	0.00605 (12)	
As2	0.60661 (4)	0.41481 (3)	0.17808 (3)	0.00626 (12)	
Al1	0.28023 (13)	0.14677 (8)	0.43819 (9)	0.00571 (19)	
Na1	0.2321 (2)	0.84727 (12)	0.63044 (15)	0.0168 (3)	
Na2	0.9037 (2)	0.91868 (11)	0.21472 (15)	0.0149 (3)	
Na3	0.6231 (2)	0.39550 (13)	0.52395 (16)	0.0201 (3)	
O1	0.7295 (3)	0.59717 (19)	0.4790 (2)	0.0126 (5)	
O2	0.9149 (3)	0.80210 (19)	0.4647 (2)	0.0091 (4)	
O3	0.4121 (3)	0.4885 (2)	0.2261 (3)	0.0131 (5)	
O4	0.2312 (3)	0.00301 (19)	0.4010 (2)	0.0102 (4)	
O5	0.0778 (3)	0.6176 (2)	0.3349 (2)	0.0113 (4)	
O6	0.5203 (3)	0.32759 (18)	0.0365 (2)	0.0098 (4)	
O7	0.7477 (3)	0.34378 (19)	0.3040 (2)	0.0123 (5)	
O8	0.7236 (3)	0.71895 (19)	0.2241 (2)	0.0116 (5)	

### Atomic displacement parameters (Å^2^)

**Table:**

	*U*^11^	*U*^22^	*U*^33^	*U*^12^	*U*^13^	*U*^23^
As1	0.00779 (17)	0.00666 (18)	0.00357 (17)	0.00099 (10)	0.00013 (11)	−0.00017 (10)
As2	0.00722 (17)	0.00686 (18)	0.00430 (18)	0.00007 (10)	−0.00108 (11)	0.00082 (10)
Al1	0.0066 (4)	0.0071 (4)	0.0032 (4)	0.0002 (3)	−0.0009 (3)	−0.0007 (3)
Na1	0.0191 (7)	0.0168 (7)	0.0155 (7)	−0.0028 (5)	0.0064 (5)	−0.0036 (6)
Na2	0.0146 (6)	0.0155 (7)	0.0145 (7)	0.0011 (5)	0.0012 (5)	0.0002 (5)
Na3	0.0125 (6)	0.0265 (8)	0.0205 (7)	0.0017 (6)	−0.0013 (5)	−0.0017 (6)
O1	0.0131 (10)	0.0150 (11)	0.0101 (11)	−0.0012 (9)	0.0033 (8)	0.0031 (9)
O2	0.0114 (10)	0.0079 (9)	0.0080 (10)	0.0004 (8)	0.0011 (8)	−0.0045 (9)
O3	0.0116 (10)	0.0130 (11)	0.0154 (11)	0.0022 (9)	0.0053 (9)	−0.0017 (9)
O4	0.0107 (10)	0.0091 (10)	0.0108 (10)	0.0014 (8)	0.0016 (8)	−0.0018 (9)
O5	0.0109 (10)	0.0119 (11)	0.0116 (11)	0.0042 (9)	0.0037 (8)	−0.0018 (9)
O6	0.0110 (10)	0.0113 (11)	0.0065 (10)	−0.0002 (8)	−0.0017 (8)	−0.0016 (8)
O7	0.0155 (11)	0.0129 (11)	0.0076 (10)	0.0010 (9)	−0.0034 (8)	0.0033 (9)
O8	0.0170 (11)	0.0123 (11)	0.0043 (10)	0.0052 (9)	−0.0037 (8)	−0.0003 (8)

### Geometric parameters (Å, º)

**Table:**

Al1—O2^i^	1.771 (2)	Na3—O1^i^	2.324 (3)
Al1—O4	1.755 (2)	Na3—O7	2.403 (3)
Al1—O6^ii^	1.769 (2)	Na3—O1	2.531 (3)
Al1—O8^iii^	1.755 (2)	Na3—O6^ii^	2.725 (3)
As1—O1	1.654 (2)	Na3—O3^i^	2.767 (3)
As1—O2	1.732 (2)	O1—Na3^i^	2.324 (3)
As1—O5^iv^	1.649 (2)	O1—Na2^vi^	2.399 (3)
As2—O3	1.655 (2)	O2—Al1^i^	1.771 (2)
As2—O4^v^	1.723 (2)	O2—Na1^iv^	2.525 (3)
As2—O6	1.734 (2)	O3—Na2^iii^	2.366 (3)
As2—O7	1.655 (2)	O3—Na1^ix^	2.396 (3)
Na1—O5^vi^	2.324 (3)	O3—Na3^i^	2.767 (3)
Na1—O7^i^	2.338 (3)	O4—As2^iii^	1.723 (2)
Na1—O3^vi^	2.396 (3)	O4—Na2^xii^	2.811 (3)
Na1—O6^v^	2.410 (3)	O4—Na1^xiii^	2.845 (3)
Na1—O2^vii^	2.525 (3)	O5—As1^vii^	1.649 (2)
Na1—O4^viii^	2.845 (3)	O5—Na3^i^	2.259 (3)
Na2—O3^v^	2.366 (3)	O5—Na1^ix^	2.324 (3)
Na2—O1^ix^	2.399 (3)	O5—Na2^iii^	2.400 (3)
Na2—O5^v^	2.400 (3)	O6—Al1^xiv^	1.769 (2)
Na2—O7^x^	2.488 (3)	O6—Na1^iii^	2.410 (3)
Na2—O8	2.646 (3)	O6—Na3^xiv^	2.725 (3)
Na2—O2	2.732 (3)	O7—Na1^i^	2.338 (3)
Na2—O4^xi^	2.811 (3)	O7—Na2^xv^	2.488 (3)
Na3—O5^i^	2.259 (3)	O8—Al1^v^	1.755 (2)
			
O5^iv^—As1—O1	116.68 (12)	O1^ix^—Na2—O5^v^	85.63 (9)
O5^iv^—As1—O8	108.15 (11)	O3^v^—Na2—O7^x^	170.45 (10)
O1—As1—O8	112.63 (11)	O1^ix^—Na2—O7^x^	104.75 (9)
O5^iv^—As1—O2	110.31 (11)	O5^v^—Na2—O7^x^	105.41 (9)
O1—As1—O2	106.86 (11)	O3^v^—Na2—O8	83.77 (9)
O8—As1—O2	101.07 (11)	O1^ix^—Na2—O8	78.02 (8)
O3—As2—O7	117.25 (12)	O5^v^—Na2—O8	155.65 (9)
O3—As2—O4^v^	109.92 (11)	O7^x^—Na2—O8	96.20 (8)
O7—As2—O4^v^	107.04 (11)	O3^v^—Na2—O2	84.72 (8)
O3—As2—O6	109.11 (11)	O1^ix^—Na2—O2	136.89 (9)
O7—As2—O6	111.39 (11)	O5^v^—Na2—O2	131.71 (9)
O4^v^—As2—O6	100.85 (11)	O7^x^—Na2—O2	87.06 (8)
O8^iii^—Al1—O4	108.02 (11)	O8—Na2—O2	59.34 (7)
O8^iii^—Al1—O6^ii^	107.51 (11)	O3^v^—Na2—O4^xi^	111.25 (9)
O4—Al1—O6^ii^	113.86 (11)	O1^ix^—Na2—O4^xi^	149.78 (9)
O8^iii^—Al1—O2^i^	110.53 (11)	O5^v^—Na2—O4^xi^	73.97 (8)
O4—Al1—O2^i^	107.78 (11)	O7^x^—Na2—O4^xi^	61.34 (7)
O6^ii^—Al1—O2^i^	109.15 (11)	O8—Na2—O4^xi^	127.61 (8)
O5^vi^—Na1—O7^i^	88.17 (9)	O2—Na2—O4^xi^	72.02 (7)
O5^vi^—Na1—O3^vi^	77.64 (9)	O5^i^—Na3—O1^i^	144.58 (11)
O7^i^—Na1—O3^vi^	131.97 (10)	O5^i^—Na3—O7	97.69 (9)
O5^vi^—Na1—O6^v^	163.14 (10)	O1^i^—Na3—O7	116.07 (10)
O7^i^—Na1—O6^v^	93.36 (9)	O5^i^—Na3—O1	85.63 (9)
O3^vi^—Na1—O6^v^	88.99 (9)	O1^i^—Na3—O1	105.03 (9)
O5^vi^—Na1—O2^vii^	98.35 (9)	O7—Na3—O1	87.95 (9)
O7^i^—Na1—O2^vii^	88.83 (9)	O5^i^—Na3—O6^ii^	96.48 (9)
O3^vi^—Na1—O2^vii^	138.21 (10)	O1^i^—Na3—O6^ii^	77.46 (8)
O6^v^—Na1—O2^vii^	98.46 (8)	O7—Na3—O6^ii^	84.47 (9)
O5^vi^—Na1—O4^viii^	123.90 (9)	O1—Na3—O6^ii^	172.34 (10)
O7^i^—Na1—O4^viii^	145.14 (9)	O5^i^—Na3—O3^i^	71.35 (8)
O3^vi^—Na1—O4^viii^	73.99 (8)	O1^i^—Na3—O3^i^	79.09 (9)
O6^v^—Na1—O4^viii^	60.27 (7)	O7—Na3—O3^i^	159.60 (10)
O2^vii^—Na1—O4^viii^	74.45 (8)	O1—Na3—O3^i^	74.37 (8)
O3^v^—Na2—O1^ix^	84.62 (9)	O6^ii^—Na3—O3^i^	113.29 (9)
O3^v^—Na2—O5^v^	76.78 (9)		

Symmetry codes: (i) −*x*+1, −*y*+1, −*z*+1; (ii) *x*, −*y*+1/2, *z*+1/2; (iii) −*x*+1, *y*−1/2, −*z*+1/2; (iv) *x*+1, *y*, *z*; (v) −*x*+1, *y*+1/2, −*z*+1/2; (vi) *x*, −*y*+3/2, *z*+1/2; (vii) *x*−1, *y*, *z*; (viii) *x*, *y*+1, *z*; (ix) *x*, −*y*+3/2, *z*−1/2; (x) −*x*+2, *y*+1/2, −*z*+1/2; (xi) *x*+1, *y*+1, *z*; (xii) *x*−1, *y*−1, *z*; (xiii) *x*, *y*−1, *z*; (xiv) *x*, −*y*+1/2, *z*−1/2; (xv) −*x*+2, *y*−1/2, −*z*+1/2.
